# Draft Genome Sequences of Five Historical Bacillus anthracis Strains

**DOI:** 10.1128/MRA.01130-19

**Published:** 2020-01-02

**Authors:** Daniel D. Sommer, Shashikala Ratnayake, Diana Radune, Kisha Parker, Sana Enke, Tracy M. Ferguson, Marie Lovett, Adam Mallonee, Zach Rae, M. J. Rosovitz, Lynn F. Diviak, Mary Beth Friss, Joy P. Klubnik, Kathy H. Fronda, Gregory P. Horn, Thomas E. Blank, Robert K. Pope, Philip C. Hanna, Nicholas H. Bergman, Adam L. Bazinet

**Affiliations:** a National Biodefense Analysis and Countermeasures Center, Fort Detrick, Maryland, USA; b University of Michigan Medical School, Ann Arbor, Michigan, USA; University of Arizona

## Abstract

Bacillus anthracis is the causative agent of anthrax, a disease of livestock, wildlife, and humans. Here, we present the draft genome sequences of five historical B. anthracis strains that were preserved as lyophilates in glass vials for decades.

## ANNOUNCEMENT

Eight glass vials of Bacillus anthracis produced at ATCC from 1962 to 1988 were opened, and the lyophilized contents were resuspended in tryptic soy broth (TSB) and cultured in TSB and on 5% sheep blood agar (SBA) at 35°C. The glass vials were originally sealed such that they would have had to be broken to be tampered with or otherwise contaminated, thus adding a degree of confidence as to the origin of the material being sequenced. Viable bacteria were not recovered from three of the vials (ATCC 10 lot number 1982-Aug-20, ATCC 938 lot number 1963-May-02, and ATCC 11949 lot number 1962-July-19). The remaining five vials (Table 1) yielded nonhemolytic Gram-positive rods that were sensitive to both penicillin and gamma phage. Frozen stocks were prepared from the cultured material. Starting from these stocks, bacteria were subcultured overnight at 35°C to form a lawn on SBA. DNA was extracted from the subcultured material using the Promega Wizard genomic DNA purification kit and filtered through a 0.1-μm spin filter. Ten percent of the DNA volume was inoculated into 10 ml of TSB and incubated at 35°C for at least 48 hours, and then 100 μl of the broth was plated to SBA and incubated at 35°C for at least 48 additional hours to confirm sterility. B. anthracis strains were handled according to Federal Select Agent Program regulations.

**TABLE 1:** Strain information and assembly statistics

ATCC no.	Lot no.	GenBank accession no.	Phylogenetic clade	Plasmid status^*a*^	No. of reads	Avg read coverage (×)	*N*_50_ (Mb)	No. of contigs
ATCC 240	1988-Sept	SAMN12620928	TEA/Pasteur	pXO1– pXO2+	8,752,370	963	1.13	23
ATCC 937	1962-July-10	SAMN12620929	TEA/WNA	pXO1– pXO2+	7,699,006	758	0.94	23
ATCC 4728	1962-May-04	SAMN12620930	TEA/Pasteur	pXO1– pXO2+	8,171,984	283	1.16	20
ATCC 6603	1963-June-23	SAMN12620931	Aust94	pXO1– pXO2+	8,551,938	749	0.60	26
ATCC 11966	1988-Sept	SAMN12620932	Vollum	pXO1+ pXO2+	10,521,400	593	0.94	32

a −, absent; +, present.

Illumina Nextera XT libraries were prepared from the extracted DNA samples using the standard Illumina protocol. The final libraries were pooled and sequenced on the Illumina HiSeq 2500 instrument, generating paired-end reads of 250 bp. For the bioinformatic analyses that followed, default parameters were used with all software programs unless otherwise noted. Reads were preprocessed before assembly using bmap_preprocess (https://github.com/bioforensics/asm_tools/) with the parameters –qual 20 and –length 75. The bmap_preprocess workflow filtered the reads using fastp version 0.19.3 (1) to ensure the minimum length (75 bp) and quality value (20) for at least 60% of the bases, estimated the genome size by building a *k*-mer profile using Jellyfish (2), and randomly downsampled the reads to an estimated 150× genome coverage. Genome assembly was performed using SPAdes version 3.12.0 (3), and genome quality assessment was performed using QUAST version 4.6.3 (4). All reads were mapped back to the assembly using Bowtie 2 (5) to determine the average genome coverage values (Table 1). The assembled genomes had an estimated size of 5.3 to 5.5 Mb with ≈35% GC content. The presence or absence of pXO plasmids was determined by aligning assembled contigs to reference pXO sequences (Ames Ancestor) using MUMmer (NUCmer) version 3.1 (6). In our analysis, four strains were found to lack the pXO1 plasmid (NC_007322); all strains contained the pXO2 plasmid (NC_007323) (Table 1). Core genome alignment and phylogenetic analysis of the five ATCC strains together with all publicly available B. anthracis genomes was performed using Parsnp version 1.0 (7) and RAxML version 8.2.12 (8) with the GTR+Gamma+I substitution model (-m GTRGAMMAI). The phylogenetic results were compared to previously published B. anthracis phylogenies (9) to determine the phylogenetic clade label for each strain (Table 1).

The provenance of the five strains was previously described (10, 11). ATCC 240 and ATCC 937 were part of the ATCC collection prior to 1931 (12), and ATCC 4728 and ATCC 6603 were part of the collection by 1952 (11). ATCC 4728 was previously sequenced as Smith 1013 and A0157 (NCBI nucleotide accession numbers JNOD00000000, CP010342, and CP010343; 13). Further analysis of the sequence of ATCC 11966, a laboratory-derived nonproteolytic mutant of the Vollum strain (14), may identify mutations affecting the expression or activity of proteases.

### Data availability.

This whole-genome shotgun project has been deposited in DDBJ/ENA/GenBank under the BioSample accession numbers SAMN12620928, SAMN12620929, SAMN12620930, SAMN12620931, and SAMN12620932. The raw Illumina paired-end sequencing reads have been deposited in the Sequence Read Archive under the accession numbers SRR10019497, SRR10019498, SRR10019499, SRR10019500, and SRR10019501.
